# Supplementary material for: Pyruvate Dehydrogenase Kinase 1 inhibition mediated oxidative phosphorylation enhancement in cartilage promotes osteoarthritis progression
Source: BMC Musculoskelet Disord. 2023 Jul 20;24:597. doi: 10.1186/s12891-023-06585-6 (PMC10357736; doi:10.1186/s12891-023-06585-6)
Supplement: Supplementary file 1 — Additional file 1. [file 12891_2023_6585_MOESM1_ESM.zip › MF.Gsea.1653627316242/GOMF_NUCLEASE_ACTIVITY.html]

Details for gene set GOMF\_NUCLEASE\_ACTIVITY[GSEA]

|  || Dataset | 55457\_collapsed\_to\_symbols.cls.cls #OA\_versus\_normal.cls.cls #OA\_versus\_normal\_repos |
| Phenotype | cls.cls#OA\_versus\_normal\_repos |
| Upregulated in class | OA |
| GeneSet | GOMF\_NUCLEASE\_ACTIVITY |
| Enrichment Score (ES) | 0.22465459 |
| Normalized Enrichment Score (NES) | 1.1225055 |
| Nominal p-value | 0.18387097 |
| FDR q-value | 0.6546082 |
| FWER p-Value | 1.0 |
Table: GSEA Results Summary

  

Fig 1: Enrichment plot: GOMF\_NUCLEASE\_ACTIVITY      
 Profile of the Running ES Score & Positions of GeneSet Members on the Rank Ordered List

  

| SYMBOL | TITLE | RANK IN GENE LIST | RANK METRIC SCORE | RUNNING ES | CORE ENRICHMENT || 1 | PAN2 | poly(A) specific ribonuclease subunit PAN2 [Source:HGNC Symbol;Acc:HGNC:20074] | 190 | 0.828 | 0.0027 | Yes |
| 2 | RPP30 | ribonuclease P/MRP subunit p30 [Source:HGNC Symbol;Acc:HGNC:17688] | 265 | 0.777 | 0.0133 | Yes |
| 3 | TSN | translin [Source:HGNC Symbol;Acc:HGNC:12379] | 372 | 0.717 | 0.0202 | Yes |
| 4 | TREX1 | three prime repair exonuclease 1 [Source:HGNC Symbol;Acc:HGNC:12269] | 393 | 0.709 | 0.0337 | Yes |
| 5 | OGG1 | 8-oxoguanine DNA glycosylase [Source:HGNC Symbol;Acc:HGNC:8125] | 453 | 0.681 | 0.0435 | Yes |
| 6 | POP5 | "POP5 homolog, ribonuclease P/MRP subunit [Source:HGNC Symbol;Acc:HGNC:17689]" | 494 | 0.662 | 0.0544 | Yes |
| 7 | RNASET2 | ribonuclease T2 [Source:HGNC Symbol;Acc:HGNC:21686] | 522 | 0.651 | 0.0661 | Yes |
| 8 | ASTE1 | asteroid homolog 1 [Source:HGNC Symbol;Acc:HGNC:25021] | 768 | 0.576 | 0.0591 | Yes |
| 9 | POLD1 | "DNA polymerase delta 1, catalytic subunit [Source:HGNC Symbol;Acc:HGNC:9175]" | 811 | 0.566 | 0.0678 | Yes |
| 10 | RPP14 | ribonuclease P/MRP subunit p14 [Source:HGNC Symbol;Acc:HGNC:30327] | 835 | 0.559 | 0.0779 | Yes |
| 11 | PPP1R8 | protein phosphatase 1 regulatory subunit 8 [Source:HGNC Symbol;Acc:HGNC:9296] | 885 | 0.547 | 0.0856 | Yes |
| 12 | REXO5 | RNA exonuclease 5 [Source:HGNC Symbol;Acc:HGNC:24661] | 972 | 0.524 | 0.0900 | Yes |
| 13 | RAD1 | RAD1 checkpoint DNA exonuclease [Source:HGNC Symbol;Acc:HGNC:9806] | 987 | 0.521 | 0.0999 | Yes |
| 14 | PRORP | protein only RNase P catalytic subunit [Source:HGNC Symbol;Acc:HGNC:19958] | 1013 | 0.515 | 0.1089 | Yes |
| 15 | POP4 | "POP4 homolog, ribonuclease P/MRP subunit [Source:HGNC Symbol;Acc:HGNC:30081]" | 1015 | 0.514 | 0.1197 | Yes |
| 16 | RAD51C | RAD51 paralog C [Source:HGNC Symbol;Acc:HGNC:9820] | 1027 | 0.512 | 0.1297 | Yes |
| 17 | DCLRE1A | DNA cross-link repair 1A [Source:HGNC Symbol;Acc:HGNC:17660] | 1073 | 0.500 | 0.1368 | Yes |
| 18 | RNASEH2A | ribonuclease H2 subunit A [Source:HGNC Symbol;Acc:HGNC:18518] | 1102 | 0.495 | 0.1451 | Yes |
| 19 | MYG1 | MYG1 exonuclease [Source:HGNC Symbol;Acc:HGNC:17590] | 1121 | 0.492 | 0.1541 | Yes |
| 20 | DNASE2 | "deoxyribonuclease 2, lysosomal [Source:HGNC Symbol;Acc:HGNC:2960]" | 1133 | 0.489 | 0.1636 | Yes |
| 21 | POP7 | "POP7 homolog, ribonuclease P/MRP subunit [Source:HGNC Symbol;Acc:HGNC:19949]" | 1337 | 0.452 | 0.1573 | Yes |
| 22 | DCLRE1B | DNA cross-link repair 1B [Source:HGNC Symbol;Acc:HGNC:17641] | 1521 | 0.426 | 0.1520 | Yes |
| 23 | PNKP | polynucleotide kinase 3'-phosphatase [Source:HGNC Symbol;Acc:HGNC:9154] | 1662 | 0.404 | 0.1496 | Yes |
| 24 | CNOT7 | CCR4-NOT transcription complex subunit 7 [Source:HGNC Symbol;Acc:HGNC:14101] | 1676 | 0.402 | 0.1571 | Yes |
| 25 | ERCC4 | "ERCC excision repair 4, endonuclease catalytic subunit [Source:HGNC Symbol;Acc:HGNC:3436]" | 1696 | 0.400 | 0.1641 | Yes |
| 26 | XRCC3 | X-ray repair cross complementing 3 [Source:HGNC Symbol;Acc:HGNC:12830] | 1902 | 0.372 | 0.1559 | Yes |
| 27 | DXO | decapping exoribonuclease [Source:HGNC Symbol;Acc:HGNC:2992] | 1960 | 0.366 | 0.1592 | Yes |
| 28 | EXOSC7 | exosome component 7 [Source:HGNC Symbol;Acc:HGNC:28112] | 1969 | 0.364 | 0.1663 | Yes |
| 29 | ANG | angiogenin [Source:HGNC Symbol;Acc:HGNC:483] | 2009 | 0.360 | 0.1708 | Yes |
| 30 | DROSHA | drosha ribonuclease III [Source:HGNC Symbol;Acc:HGNC:17904] | 2054 | 0.356 | 0.1749 | Yes |
| 31 | RNASE6 | ribonuclease A family member k6 [Source:HGNC Symbol;Acc:HGNC:10048] | 2059 | 0.355 | 0.1822 | Yes |
| 32 | DNASE1L1 | deoxyribonuclease 1 like 1 [Source:HGNC Symbol;Acc:HGNC:2957] | 2079 | 0.353 | 0.1881 | Yes |
| 33 | PLD3 | phospholipase D family member 3 [Source:HGNC Symbol;Acc:HGNC:17158] | 2086 | 0.351 | 0.1951 | Yes |
| 34 | DBR1 | debranching RNA lariats 1 [Source:HGNC Symbol;Acc:HGNC:15594] | 2091 | 0.351 | 0.2023 | Yes |
| 35 | EXD2 | exonuclease 3'-5' domain containing 2 [Source:HGNC Symbol;Acc:HGNC:20217] | 2166 | 0.342 | 0.2037 | Yes |
| 36 | XRCC1 | X-ray repair cross complementing 1 [Source:HGNC Symbol;Acc:HGNC:12828] | 2182 | 0.340 | 0.2097 | Yes |
| 37 | ENDOD1 | endonuclease domain containing 1 [Source:HGNC Symbol;Acc:HGNC:29129] | 2242 | 0.334 | 0.2122 | Yes |
| 38 | DCPS | "decapping enzyme, scavenger [Source:HGNC Symbol;Acc:HGNC:29812]" | 2570 | 0.295 | 0.1928 | Yes |
| 39 | LACTB2 | lactamase beta 2 [Source:HGNC Symbol;Acc:HGNC:18512] | 2604 | 0.290 | 0.1964 | Yes |
| 40 | ANGEL2 | angel homolog 2 [Source:HGNC Symbol;Acc:HGNC:30534] | 2608 | 0.290 | 0.2023 | Yes |
| 41 | DCP2 | decapping mRNA 2 [Source:HGNC Symbol;Acc:HGNC:24452] | 2659 | 0.284 | 0.2044 | Yes |
| 42 | NYNRIN | NYN domain and retroviral integrase containing [Source:HGNC Symbol;Acc:HGNC:20165] | 2691 | 0.280 | 0.2079 | Yes |
| 43 | APTX | aprataxin [Source:HGNC Symbol;Acc:HGNC:15984] | 2702 | 0.279 | 0.2130 | Yes |
| 44 | ERCC1 | "ERCC excision repair 1, endonuclease non-catalytic subunit [Source:HGNC Symbol;Acc:HGNC:3433]" | 2948 | 0.255 | 0.1992 | Yes |
| 45 | REXO4 | "REX4 homolog, 3'-5' exonuclease [Source:HGNC Symbol;Acc:HGNC:12820]" | 2953 | 0.255 | 0.2043 | Yes |
| 46 | RNASE2 | ribonuclease A family member 2 [Source:HGNC Symbol;Acc:HGNC:10045] | 2976 | 0.252 | 0.2079 | Yes |
| 47 | PARN | poly(A)-specific ribonuclease [Source:HGNC Symbol;Acc:HGNC:8609] | 3100 | 0.240 | 0.2034 | Yes |
| 48 | ELAC1 | elaC ribonuclease Z 1 [Source:HGNC Symbol;Acc:HGNC:14197] | 3108 | 0.239 | 0.2079 | Yes |
| 49 | SETMAR | SET domain and mariner transposase fusion gene [Source:HGNC Symbol;Acc:HGNC:10762] | 3216 | 0.229 | 0.2043 | Yes |
| 50 | NTHL1 | nth like DNA glycosylase 1 [Source:HGNC Symbol;Acc:HGNC:8028] | 3219 | 0.228 | 0.2090 | Yes |
| 51 | SLX1B | "SLX1 homolog B, structure-specific endonuclease subunit [Source:HGNC Symbol;Acc:HGNC:28748]" | 3229 | 0.227 | 0.2131 | Yes |
| 52 | DDX1 | DEAD-box helicase 1 [Source:HGNC Symbol;Acc:HGNC:2734] | 3305 | 0.221 | 0.2119 | Yes |
| 53 | MRPL44 | mitochondrial ribosomal protein L44 [Source:HGNC Symbol;Acc:HGNC:16650] | 3306 | 0.220 | 0.2166 | Yes |
| 54 | ENPP1 | ectonucleotide pyrophosphatase/phosphodiesterase 1 [Source:HGNC Symbol;Acc:HGNC:3356] | 3392 | 0.212 | 0.2145 | Yes |
| 55 | AGO1 | argonaute RISC component 1 [Source:HGNC Symbol;Acc:HGNC:3262] | 3430 | 0.208 | 0.2160 | Yes |
| 56 | KHNYN | KH and NYN domain containing [Source:HGNC Symbol;Acc:HGNC:20166] | 3457 | 0.206 | 0.2183 | Yes |
| 57 | EXO5 | exonuclease 5 [Source:HGNC Symbol;Acc:HGNC:26115] | 3487 | 0.204 | 0.2203 | Yes |
| 58 | RIDA | reactive intermediate imine deaminase A homolog [Source:HGNC Symbol;Acc:HGNC:16897] | 3488 | 0.203 | 0.2247 | Yes |
| 59 | TSEN34 | tRNA splicing endonuclease subunit 34 [Source:HGNC Symbol;Acc:HGNC:15506] | 3617 | 0.192 | 0.2187 | No |
| 60 | RBBP8 | "RB binding protein 8, endonuclease [Source:HGNC Symbol;Acc:HGNC:9891]" | 3739 | 0.182 | 0.2131 | No |
| 61 | CNOT1 | CCR4-NOT transcription complex subunit 1 [Source:HGNC Symbol;Acc:HGNC:7877] | 3842 | 0.172 | 0.2087 | No |
| 62 | SND1 | staphylococcal nuclease and tudor domain containing 1 [Source:HGNC Symbol;Acc:HGNC:30646] | 3897 | 0.165 | 0.2080 | No |
| 63 | ENDOG | endonuclease G [Source:HGNC Symbol;Acc:HGNC:3346] | 4091 | 0.147 | 0.1959 | No |
| 64 | TDP2 | tyrosyl-DNA phosphodiesterase 2 [Source:HGNC Symbol;Acc:HGNC:17768] | 4124 | 0.144 | 0.1965 | No |
| 65 | MUS81 | MUS81 structure-specific endonuclease subunit [Source:HGNC Symbol;Acc:HGNC:29814] | 4139 | 0.143 | 0.1984 | No |
| 66 | DIS3 | "DIS3 homolog, exosome endoribonuclease and 3'-5' exoribonuclease [Source:HGNC Symbol;Acc:HGNC:20604]" | 4187 | 0.139 | 0.1977 | No |
| 67 | RPP21 | ribonuclease P/MRP subunit p21 [Source:HGNC Symbol;Acc:HGNC:21300] | 4269 | 0.131 | 0.1941 | No |
| 68 | EXOSC5 | exosome component 5 [Source:HGNC Symbol;Acc:HGNC:24662] | 4295 | 0.128 | 0.1948 | No |
| 69 | RNASE4 | ribonuclease A family member 4 [Source:HGNC Symbol;Acc:HGNC:10047] | 4353 | 0.124 | 0.1930 | No |
| 70 | ERCC5 | "ERCC excision repair 5, endonuclease [Source:HGNC Symbol;Acc:HGNC:3437]" | 4380 | 0.122 | 0.1936 | No |
| 71 | TEFM | "transcription elongation factor, mitochondrial [Source:HGNC Symbol;Acc:HGNC:26223]" | 4447 | 0.117 | 0.1909 | No |
| 72 | ELAC2 | elaC ribonuclease Z 2 [Source:HGNC Symbol;Acc:HGNC:14198] | 4532 | 0.110 | 0.1866 | No |
| 73 | APEX2 | apurinic/apyrimidinic endodeoxyribonuclease 2 [Source:HGNC Symbol;Acc:HGNC:17889] | 4551 | 0.109 | 0.1875 | No |
| 74 | CNOT8 | CCR4-NOT transcription complex subunit 8 [Source:HGNC Symbol;Acc:HGNC:9207] | 4581 | 0.106 | 0.1875 | No |
| 75 | ERI3 | ERI1 exoribonuclease family member 3 [Source:HGNC Symbol;Acc:HGNC:17276] | 4627 | 0.102 | 0.1861 | No |
| 76 | TDP1 | tyrosyl-DNA phosphodiesterase 1 [Source:HGNC Symbol;Acc:HGNC:18884] | 4832 | 0.085 | 0.1719 | No |
| 77 | FEN1 | flap structure-specific endonuclease 1 [Source:HGNC Symbol;Acc:HGNC:3650] | 5212 | 0.056 | 0.1434 | No |
| 78 | RNASE3 | ribonuclease A family member 3 [Source:HGNC Symbol;Acc:HGNC:10046] | 5463 | 0.036 | 0.1245 | No |
| 79 | INTS11 | integrator complex subunit 11 [Source:HGNC Symbol;Acc:HGNC:26052] | 5544 | 0.029 | 0.1189 | No |
| 80 | ERN1 | endoplasmic reticulum to nucleus signaling 1 [Source:HGNC Symbol;Acc:HGNC:3449] | 5658 | 0.019 | 0.1104 | No |
| 81 | EXOSC4 | exosome component 4 [Source:HGNC Symbol;Acc:HGNC:18189] | 5693 | 0.016 | 0.1081 | No |
| 82 | CNOT2 | CCR4-NOT transcription complex subunit 2 [Source:HGNC Symbol;Acc:HGNC:7878] | 5740 | 0.012 | 0.1047 | No |
| 83 | ANGEL1 | angel homolog 1 [Source:HGNC Symbol;Acc:HGNC:19961] | 5748 | 0.012 | 0.1045 | No |
| 84 | SAMHD1 | SAM and HD domain containing deoxynucleoside triphosphate triphosphohydrolase 1 [Source:HGNC Symbol;Acc:HGNC:15925] | 5790 | 0.009 | 0.1014 | No |
| 85 | USB1 | U6 snRNA biogenesis phosphodiesterase 1 [Source:HGNC Symbol;Acc:HGNC:25792] | 5797 | 0.008 | 0.1011 | No |
| 86 | RNASE1 | "ribonuclease A family member 1, pancreatic [Source:HGNC Symbol;Acc:HGNC:10044]" | 5945 | -0.001 | 0.0896 | No |
| 87 | N4BP1 | NEDD4 binding protein 1 [Source:HGNC Symbol;Acc:HGNC:29850] | 6040 | -0.007 | 0.0824 | No |
| 88 | DNASE1L3 | deoxyribonuclease 1 like 3 [Source:HGNC Symbol;Acc:HGNC:2959] | 6091 | -0.012 | 0.0787 | No |
| 89 | PIWIL1 | piwi like RNA-mediated gene silencing 1 [Source:HGNC Symbol;Acc:HGNC:9007] | 6144 | -0.016 | 0.0750 | No |
| 90 | SMG6 | SMG6 nonsense mediated mRNA decay factor [Source:HGNC Symbol;Acc:HGNC:17809] | 6175 | -0.019 | 0.0730 | No |
| 91 | ISG20 | interferon stimulated exonuclease gene 20 [Source:HGNC Symbol;Acc:HGNC:6130] | 6212 | -0.022 | 0.0707 | No |
| 92 | POP1 | "POP1 homolog, ribonuclease P/MRP subunit [Source:HGNC Symbol;Acc:HGNC:30129]" | 6261 | -0.025 | 0.0674 | No |
| 93 | FAN1 | FANCD2 and FANCI associated nuclease 1 [Source:HGNC Symbol;Acc:HGNC:29170] | 6323 | -0.030 | 0.0633 | No |
| 94 | NME1 | NME/NM23 nucleoside diphosphate kinase 1 [Source:HGNC Symbol;Acc:HGNC:7849] | 6328 | -0.031 | 0.0636 | No |
| 95 | TSEN2 | tRNA splicing endonuclease subunit 2 [Source:HGNC Symbol;Acc:HGNC:28422] | 6331 | -0.031 | 0.0641 | No |
| 96 | RAD50 | RAD50 double strand break repair protein [Source:HGNC Symbol;Acc:HGNC:9816] | 6474 | -0.040 | 0.0538 | No |
| 97 | TATDN2 | TatD DNase domain containing 2 [Source:HGNC Symbol;Acc:HGNC:28988] | 6575 | -0.047 | 0.0470 | No |
| 98 | RAG1 | recombination activating 1 [Source:HGNC Symbol;Acc:HGNC:9831] | 6806 | -0.063 | 0.0303 | No |
| 99 | RPP38 | ribonuclease P/MRP subunit p38 [Source:HGNC Symbol;Acc:HGNC:30329] | 6814 | -0.064 | 0.0311 | No |
| 100 | MAP1S | microtubule associated protein 1S [Source:HGNC Symbol;Acc:HGNC:15715] | 6848 | -0.067 | 0.0299 | No |
| 101 | PLSCR1 | phospholipid scramblase 1 [Source:HGNC Symbol;Acc:HGNC:9092] | 6917 | -0.073 | 0.0262 | No |
| 102 | ERN2 | endoplasmic reticulum to nucleus signaling 2 [Source:HGNC Symbol;Acc:HGNC:16942] | 7565 | -0.124 | -0.0220 | No |
| 103 | TSNAX | translin associated factor X [Source:HGNC Symbol;Acc:HGNC:12380] | 7606 | -0.127 | -0.0224 | No |
| 104 | DNASE2B | deoxyribonuclease 2 beta [Source:HGNC Symbol;Acc:HGNC:28875] | 7840 | -0.144 | -0.0376 | No |
| 105 | EXO1 | exonuclease 1 [Source:HGNC Symbol;Acc:HGNC:3511] | 8088 | -0.162 | -0.0536 | No |
| 106 | TREX2 | three prime repair exonuclease 2 [Source:HGNC Symbol;Acc:HGNC:12270] | 8149 | -0.167 | -0.0547 | No |
| 107 | PMS2 | "PMS1 homolog 2, mismatch repair system component [Source:HGNC Symbol;Acc:HGNC:9122]" | 8172 | -0.169 | -0.0529 | No |
| 108 | PDE12 | phosphodiesterase 12 [Source:HGNC Symbol;Acc:HGNC:25386] | 8306 | -0.178 | -0.0595 | No |
| 109 | RPP40 | ribonuclease P/MRP subunit p40 [Source:HGNC Symbol;Acc:HGNC:20992] | 8318 | -0.179 | -0.0566 | No |
| 110 | EXOSC10 | exosome component 10 [Source:HGNC Symbol;Acc:HGNC:9138] | 8404 | -0.186 | -0.0593 | No |
| 111 | ENPP2 | ectonucleotide pyrophosphatase/phosphodiesterase 2 [Source:HGNC Symbol;Acc:HGNC:3357] | 8442 | -0.189 | -0.0582 | No |
| 112 | EXOSC8 | exosome component 8 [Source:HGNC Symbol;Acc:HGNC:17035] | 8554 | -0.198 | -0.0627 | No |
| 113 | APEX1 | apurinic/apyrimidinic endodeoxyribonuclease 1 [Source:HGNC Symbol;Acc:HGNC:587] | 8767 | -0.213 | -0.0748 | No |
| 114 | ZC3H12A | zinc finger CCCH-type containing 12A [Source:HGNC Symbol;Acc:HGNC:26259] | 9030 | -0.234 | -0.0904 | No |
| 115 | REXO2 | RNA exonuclease 2 [Source:HGNC Symbol;Acc:HGNC:17851] | 9390 | -0.263 | -0.1130 | No |
| 116 | EXD3 | exonuclease 3'-5' domain containing 3 [Source:HGNC Symbol;Acc:HGNC:26023] | 9474 | -0.272 | -0.1137 | No |
| 117 | RCL1 | RNA terminal phosphate cyclase like 1 [Source:HGNC Symbol;Acc:HGNC:17687] | 9586 | -0.282 | -0.1165 | No |
| 118 | RAD9A | RAD9 checkpoint clamp component A [Source:HGNC Symbol;Acc:HGNC:9827] | 9757 | -0.297 | -0.1235 | No |
| 119 | RNASEH1 | ribonuclease H1 [Source:HGNC Symbol;Acc:HGNC:18466] | 9764 | -0.297 | -0.1177 | No |
| 120 | PELO | pelota mRNA surveillance and ribosome rescue factor [Source:HGNC Symbol;Acc:HGNC:8829] | 9806 | -0.302 | -0.1145 | No |
| 121 | RNASEL | ribonuclease L [Source:HGNC Symbol;Acc:HGNC:10050] | 9965 | -0.317 | -0.1201 | No |
| 122 | WRN | WRN RecQ like helicase [Source:HGNC Symbol;Acc:HGNC:12791] | 10010 | -0.323 | -0.1167 | No |
| 123 | AZGP1 | "alpha-2-glycoprotein 1, zinc-binding [Source:HGNC Symbol;Acc:HGNC:910]" | 10069 | -0.328 | -0.1143 | No |
| 124 | EXOG | exo/endonuclease G [Source:HGNC Symbol;Acc:HGNC:3347] | 10266 | -0.347 | -0.1223 | No |
| 125 | ISG20L2 | interferon stimulated exonuclease gene 20 like 2 [Source:HGNC Symbol;Acc:HGNC:25745] | 10404 | -0.361 | -0.1254 | No |
| 126 | TOE1 | "target of EGR1, exonuclease [Source:HGNC Symbol;Acc:HGNC:15954]" | 10501 | -0.373 | -0.1251 | No |
| 127 | EXOSC9 | exosome component 9 [Source:HGNC Symbol;Acc:HGNC:9137] | 10606 | -0.384 | -0.1251 | No |
| 128 | RPP25 | ribonuclease P and MRP subunit p25 [Source:HGNC Symbol;Acc:HGNC:30361] | 10621 | -0.385 | -0.1180 | No |
| 129 | DNASE1L2 | deoxyribonuclease 1 like 2 [Source:HGNC Symbol;Acc:HGNC:2958] | 10947 | -0.424 | -0.1345 | No |
| 130 | POLE | "DNA polymerase epsilon, catalytic subunit [Source:HGNC Symbol;Acc:HGNC:9177]" | 11026 | -0.433 | -0.1314 | No |
| 131 | DNASE1 | deoxyribonuclease 1 [Source:HGNC Symbol;Acc:HGNC:2956] | 11065 | -0.440 | -0.1251 | No |
| 132 | PGBD5 | piggyBac transposable element derived 5 [Source:HGNC Symbol;Acc:HGNC:19405] | 11106 | -0.444 | -0.1188 | No |
| 133 | MBD4 | "methyl-CpG binding domain 4, DNA glycosylase [Source:HGNC Symbol;Acc:HGNC:6919]" | 11114 | -0.445 | -0.1099 | No |
| 134 | G3BP1 | G3BP stress granule assembly factor 1 [Source:HGNC Symbol;Acc:HGNC:30292] | 11117 | -0.445 | -0.1006 | No |
| 135 | CNOT6 | CCR4-NOT transcription complex subunit 6 [Source:HGNC Symbol;Acc:HGNC:14099] | 11212 | -0.459 | -0.0982 | No |
| 136 | PIWIL2 | piwi like RNA-mediated gene silencing 2 [Source:HGNC Symbol;Acc:HGNC:17644] | 11219 | -0.460 | -0.0890 | No |
| 137 | RPS3 | ribosomal protein S3 [Source:HGNC Symbol;Acc:HGNC:10420] | 11245 | -0.463 | -0.0811 | No |
| 138 | AGO2 | argonaute RISC catalytic component 2 [Source:HGNC Symbol;Acc:HGNC:3263] | 11246 | -0.463 | -0.0713 | No |
| 139 | ERI2 | ERI1 exoribonuclease family member 2 [Source:HGNC Symbol;Acc:HGNC:30541] | 11320 | -0.474 | -0.0670 | No |
| 140 | MRE11 | "MRE11 homolog, double strand break repair nuclease [Source:HGNC Symbol;Acc:HGNC:7230]" | 11458 | -0.497 | -0.0672 | No |
| 141 | ENDOU | "endonuclease, poly(U) specific [Source:HGNC Symbol;Acc:HGNC:14369]" | 11468 | -0.498 | -0.0573 | No |
| 142 | LAS1L | LAS1 like ribosome biogenesis factor [Source:HGNC Symbol;Acc:HGNC:25726] | 11474 | -0.498 | -0.0472 | No |
| 143 | NOCT | nocturnin [Source:HGNC Symbol;Acc:HGNC:14254] | 11566 | -0.510 | -0.0435 | No |
| 144 | EXOSC2 | exosome component 2 [Source:HGNC Symbol;Acc:HGNC:17097] | 12100 | -0.620 | -0.0721 | No |
| 145 | AEN | apoptosis enhancing nuclease [Source:HGNC Symbol;Acc:HGNC:25722] | 12125 | -0.625 | -0.0608 | No |
| 146 | DNA2 | DNA replication helicase/nuclease 2 [Source:HGNC Symbol;Acc:HGNC:2939] | 12148 | -0.631 | -0.0491 | No |
| 147 | DICER1 | "dicer 1, ribonuclease III [Source:HGNC Symbol;Acc:HGNC:17098]" | 12215 | -0.650 | -0.0405 | No |
| 148 | POLG | "DNA polymerase gamma, catalytic subunit [Source:HGNC Symbol;Acc:HGNC:9179]" | 12369 | -0.699 | -0.0377 | No |
| 149 | DFFB | DNA fragmentation factor subunit beta [Source:HGNC Symbol;Acc:HGNC:2773] | 12420 | -0.714 | -0.0265 | No |
| 150 | AGO3 | argonaute RISC catalytic component 3 [Source:HGNC Symbol;Acc:HGNC:18421] | 12726 | -0.888 | -0.0316 | No |
| 151 | DCLRE1C | DNA cross-link repair 1C [Source:HGNC Symbol;Acc:HGNC:17642] | 12818 | -1.002 | -0.0175 | No |
| 152 | EDC3 | enhancer of mRNA decapping 3 [Source:HGNC Symbol;Acc:HGNC:26114] | 12868 | -1.123 | 0.0025 | No |
Table: GSEA details [plain text format]

  

Fig 2: GOMF\_NUCLEASE\_ACTIVITY      
 Blue-Pink O' Gram in the Space of the Analyzed GeneSet

  

Fig 3: GOMF\_NUCLEASE\_ACTIVITY: Random ES distribution      
 Gene set null distribution of ES for **GOMF\_NUCLEASE\_ACTIVITY**
